# Identifying and Engineering Bottlenecks of Autotrophic Isobutanol Formation in Recombinant *C. ljungdahlii* by Systemic Analysis

**DOI:** 10.3389/fbioe.2021.647853

**Published:** 2021-03-03

**Authors:** Maria Hermann, Attila Teleki, Sandra Weitz, Alexander Niess, Andreas Freund, Frank Robert Bengelsdorf, Peter Dürre, Ralf Takors

**Affiliations:** ^1^Institute of Biochemical Engineering, Faculty of Energy-, Process-, and Bio-Engineering, University of Stuttgart, Stuttgart, Germany; ^2^Institute of Microbiology and Biotechnology, Faculty of Natural Sciences, University of Ulm, Ulm, Germany

**Keywords:** *Clostridium ljungdahlii*, intracellular metabolite pools, synthesis gas fermentation, recombinant product formation, isobutanol

## Abstract

*Clostridium ljungdahlii* (*C. ljungdahlii*, CLJU) is natively endowed producing acetic acid, 2,3-butandiol, and ethanol consuming gas mixtures of CO_2_, CO, and H_2_ (syngas). Here, we present the syngas-based isobutanol formation using *C. ljungdahlii* harboring the recombinant amplification of the “Ehrlich” pathway that converts intracellular KIV to isobutanol. Autotrophic isobutanol production was studied analyzing two different strains in 3-L gassed and stirred bioreactors. Physiological characterization was thoroughly applied together with metabolic profiling and flux balance analysis. Thereof, KIV and pyruvate supply were identified as key “bottlenecking” precursors limiting preliminary isobutanol formation in CLJU[KAIA] to 0.02 g L^–1^. Additional blocking of valine synthesis in CLJU[KAIA]:*ilvE* increased isobutanol production by factor 6.5 finally reaching 0.13 g L^–1^. Future metabolic engineering should focus on debottlenecking NADPH availability, whereas NADH supply is already equilibrated in the current generation of strains.

## Introduction

**FIGURE 1:**
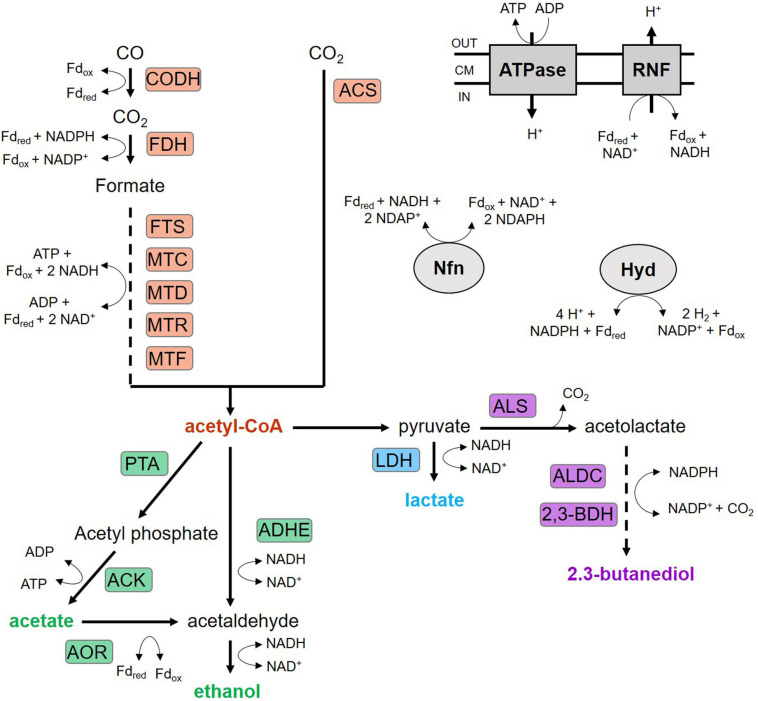
Wood-Ljungdahl pathway and product formation of *C. ljungdahlii*. ACK, acetate kinase; ACS, acetyl-CoA synthase; ADHE, aldehyde/alcohol dehydrogenase; ALDC, acetolactate decarboxylase; ALS, acetolactate synthase; AOR, aldehyde:ferredoxin oxidoreductase; CODH, CO dehydrogenase; FDH, formate dehydrogenase; FTS, formyl-THF synthetase; Hyd, electron-bifurcation hydrogenase; LDH, lactate dehydrogenase; MTC, methenyl-THF cyclohydrolase; MTD, methylene-THF dehydrogenase; MTF, methyltransferase; MTR, methylene-THF reductase; Nfn, electron-bifurcating and ferredoxin-dependent transhydrogenase; PFOR, pyruvate:ferredoxin oxidoreductase; PTA, phosphotransacetylase; PTF, phosphotransferase; RNF, Rnf complex; THF, tetrahydrofolate; 2,3-BDH: 2,3-butanediol dehydrogenase.

Isobutanol is an important commodity in the chemical, food, and pharmaceutical industries with rising global market size (Karabektas and Hosoz, 2009; Grand View Research, 2016). Furthermore, it is a promising fuel substitute showing lower vapor pressure, volatility, and hygroscopicity and higher energy density than bioethanol (Atsumi et al., 2010). Currently, the production of isobutanol is mainly based on petroleum resources. In addition, there are already several biotechnological approaches mainly based on sugars (Chen and Liao, 2016). Synthesis gas (syngas) represents a further promising substrate for biotechnological production of isobutanol as it can replace fossil-based resources and simultaneously prevent a competition with the availability of food. Syngas is a mixture mainly composed of carbon monoxide (CO), carbon dioxide (CO_2_), and hydrogen (H_2_) derived from agricultural, industrial, and municipal wastes and thus representing an inexpensive feedstock (Bengelsdorf and Dürre, 2017; Takors et al., 2018). Several anaerobic bacteria are able to metabolize syngas components via hydrogenesis, methanogenesis, or acetogenesis to a wide range of products (Latif et al., 2014; Diender et al., 2015; Takors et al., 2018). Thereof, *C. ljungdahlii* is a promising biocatalyst as it can convert autotrophically syngas, solely CO, and mixtures of CO_2_ and H_2_ to its natural products acetate, ethanol, 2,3-butanediol, and lactate (Tanner et al., 1993; Köpke et al., 2010, 2011). Its ability to fix CO and CO_2_ relies on the Wood-Ljungdahl-Pathway (WLP) that is described in detail in several excellent review articles (Drake et al., 2008; Ragsdale and Pierce, 2008; Schuchmann and Müller, 2014). Figure 1 shows a scheme of the basic metabolic pathways of syngas-fermenting *C. ljungdahlii*. In short, the WLP is a two-branched reductive pathway characterized by a stepwise reduction of CO_2_ to a methyl group (methyl branch) which subsequently is combined with CO (carbonyl branch) to acetyl-CoA, the key-precursor for biomass and products. The WLP is energy-limited as only one ATP may be generated by the conversion of acetyl-CoA to acetate. This, in turn, is needed to reduce CO_2_ in the methyl branch, leaving no net ATP formation *via* substrate-level phosphorylation. Hence, a proton gradient coupled to an H^+^-translocating ATPase is decisive for the energy provision in *C. ljungdahlii*. In this context, the membrane-bound ferredoxin:NAD oxidoreductase (Rnf complex) plays a crucial role as it couples the electron transfer from reduced ferredoxin (Fd_*red*_) to NAD^+^ to a simultaneous translocation of protons through the cell membrane (Müller et al., 2008; Tremblay et al., 2012; Hess et al., 2016). The required reducing equivalents are provided by the oxidation of CO *via* carbon monoxide dehydrogenase (CODH) or H_2_ using a bifurcating hydrogenase (Hyd) reaction (Köpke et al., 2010; Schuchmann and Müller, 2012; Buckel and Thauer, 2013; Wang et al., 2013). An electron bifurcating transhydrogenase (Nfn) reaction is also involved in the energy conservation of *C. ljungdahlii.* It catalyzes the endergonic reduction of NADP^+^ with NADH coupled to the exergonic reduction of NADP^+^ with Fd_*red*_ (Mock et al., 2015; Aklujkar et al., 2017; Liang et al., 2019). Consequently, Fd_*red*_ availability tightly links energy management, substrate composition, and product formation in *C. ljungdahlii*. In this context, we identified syngas as a suitable substrate to produce reduced alcohols, presenting the highest 2,3-butanediol formation using a batch process with *C. ljungdahlii* described so far (Hermann et al., 2020). Furthermore, *C. ljungdahlii* is genetically accessible enabling the optimized formation of natural and recombinant products *via* metabolic engineering (Huang et al., 2019; Molitor et al., 2016; Woolston et al., 2018). Weitz et al. (2021) engineered several *C. ljungdahlii* strains and successfully demonstrated isobutanol formation analyzing heterotrophic and autotrophic growth conditions by lab scale batch fermentation experiments. This study builds on the findings of Weitz et al. (2021) by investigating autotrophic syngas-based isobutanol formation applying the two recombinant strains CLJU[KAIA] and CLJU[KAIA]:*ilvE*. Based on 3-L batch cultivations in gassed stirred bioreactors, strains were physiologically characterized, thoroughly investigated *via* intracellular metabolomics, and quantified *via* Flux Balance Analysis (FBA). Thereof, promising metabolic engineering targets were derived finally yielding CLJU[KAIA]:*ilvE* which achieved 130 mg of isobutanol/L.

## Materials and Methods

A complete description of all methods below can be found in the appendix.

### Bacterial Strains, Growth Medium and Pre-culture Preparation

*Clostridium ljungdahlii* DSM 13528 (Tanner et al., 1993) was obtained from the German Collection of Microorganisms and Cell Cultures (DSMZ). The recombinant *C. ljungdahlii* strains CLJU[KAIA] and CLJU[KAIA]:*ilvE* were kindly provided by the group of Peter Dürre (Institute of Microbiology and Biotechnology, University of Ulm). Details of strain construction are described elsewhere Weitz et al. (2021). Medium and preculture seed train was described earlier (Hermann et al., 2020). The last pre-culture step was based on syngas, characterized by the same gas composition as the bioreactor substrate.

### Batch Cultivation Studies in a Stirred-Tank Reactor With Different Substrates

Anaerobic syngas-based batch cultivations were performed in a fully controlled 3-L stirred-tank bioreactor (Bioengineering, Wald, Switzerland) with an operational volume of 1.5 L. The detailed reactor equipment was previously described in Hermann et al. (2020). Temperature and pH were kept constant at 37°C and 5.9, respectively. The agitation speed of the impeller was 500 rpm during the whole cultivation process. The substrate gas was fed continuously into the reactor using one mass flow controller (Bronkhorst High-Tech B. V., Ruurlo, Netherlands) and a predefined gas mixture with a constant gassing rate of 13.2 L h^–1^. The gas composition was 55% CO, 30% H_2_, 5% CO_2_, and 10% Ar. To set anaerobic conditions, the medium-containing bioreactor was sparged with nitrogen with a gassing rate of 60 L h^–1^ applied for 2 h. Off-gas measurements guaranteed that oxygen concentrations were always below 0.01% (vv^–1^). Afterward, the medium was equilibrated with the substrate gas for 5 h. Two hours prior to inoculation of the bioreactor, sterile reducing agent was added (Tanner et al., 1993). To observe growth, extracellular product formation, and intracellular metabolite pools, samples were taken frequently during the cultivations.

All fermentations showed very similar growth and substrate uptake kinetics. Only product formation differed with respect to isobutanol production.

### Analytical Methods

#### Biomass Concentration Analysis

Cell density was determined offline *via* a UV/Visible spectrophotometer at 600 nm. A detailed description is found in Hermann et al. (2020).

#### Analysis of Extracellular Products

The extracellular formation of ethanol, acetate, 2,3-butanediol, lactate, and isobutanol was observed using an isocratic high-performance liquid chromatography (HPLC) equipped with a RI detector and a Rezex ROA-Organic Acid H^+^ column. Measuring parameters and sample preparation are described in Hermann et al. (2020).

#### LC-MS Based Analysis of Intracellular Metabolites’ Concentrations

Intracellular metabolites’ concentrations in [μmol g_*CDW*_^–1^] were quantified using an HPLC system coupled to a triple quadrupole tandem mass spectrometer (QQQ-MS/MS) equipped with an electrospray ion source. Therefore, 5 mL cell suspension each were taken periodically as triplicates in the course of the exponential growth phases of the batch cultures. Extraction and quantification of non-derivatized polar metabolites was described earlier (Teleki et al., 2015; Hermann et al., 2020). Due to their high reactivity, the analysis of α-keto acids (aKG, pyruvate, and OAA) required a preceding derivatization treatment based on the condensation of aldehyde and keto groups by phenylhydrazine (Zimmermann et al., 2014). In addition, a quantification method based on bicratic reverse phase chromatography (RPLC) with acidic mobile phase conditions was applied. For this purpose, an adapted derivatization strategy as well as the respective LC-MS/MS protocol was developed and described by Junghans et al. (2019). Therefore, for determination of the intracellular pools of pyruvate, KIV, aKG, and OAA 2.5 μL of a freshly prepared 50 mM phenylhydrazine stock solution were added to 24 μL of the metabolite extracts. Additionally, the samples were spiked with 4 μL of a defined standard mix or water and mixed with 1 μL of a 2.2 mM glyoxylate (Gxy) solution. After an incubation at room temperature for 1 h the samples were quenched with 0.5 μL of a 10%(vv^–1^) formic acid stock solution and 18 μL of acetonitrile. Gxy was considered to monitor instrumental fluctuations and the standard mix was needed for the absolute quantification of the respective α-keto acids. Based on previous measurements, the composition of the standard mix was set to 12 μM pyruvate, 1.6 μM OAA, 2 μM KIV, and 6 μM aKG. By means of different water to standard mix ratios during sample preparation internal calibration curves with four levels for each metabolite were achieved. Thus, internal calibration curves resulted from a standard quadruple addition of defined amounts of the respective metabolite standards directly to the sample.

#### Online Analysis of the Exhaust Gas

Exhaust gas measurement was performed online by mass spectrometry to determine gas uptake and production as described in Hermann et al. (2020).

#### Determination of Cell Specific Rates

For each growth phase biomass-specific substrate uptake and product formation rates were calculated by considering the exponential growth rate μ, the biomass substrate yield YX/S, or the biomass product yield YX/P, respectively. A detailed description is found in Hermann et al. (2020).

#### Determination of the Gibbs Free Energy Changes ΔG_*R*_

Gibbs free reaction energy changes ΔG_*R*_ were calculated to compare the individual processes at the energetic level as described by Villadsen et al. (2011). Corresponding calculations for the individual processes are attached in the appendix.

#### Flux Balance Analysis

Model simulations were performed based on the *Insilico* Discovery^TM^ platform using the previously reconstructed and described model rSMM (Hermann et al., 2020), which was supplemented by a formate-H_2_ lyase like reaction (Wang et al., 2013) and recombinant isobutanol formation. This model is characterized by a constant growth-associated maintenance (GAM) value of 46.7 mmol ATP g_*CDW*_^–1^ (Nagarajan et al., 2013) and the invariable non-growth-associated maintenance value (NGAM) of 5 mmol (g_*CDW*_^∗^h)^–1^. For NGAM estimation the mean maintenance cost identified for the closely related acetogen *Clostridium autoethanogenum* growing on different gaseous substrates was considered (Valgepea et al., 2018; Heffernan et al., 2020). Further assumption and characteristics of the model are described in Hermann et al. (2020). As the degree of freedom exceeds the maximal number of quantifiable fluxes, FBA was used to investigate the intracellular flux distribution (Schilling et al., 2000; Orth et al., 2010). Maximization of biomass production was set as objective function, while all experimentally determined product formation and substrate uptake rates were used to constrain the solution space (O’Brien et al., 2015). Further details of the applied FBA method are found in Hermann et al. (2020).

## Results

### Syngas-Based Batch Cultivation of CLJU[WT]

#### Growth, Product Formation and Substrate Uptake

**FIGURE 2:**
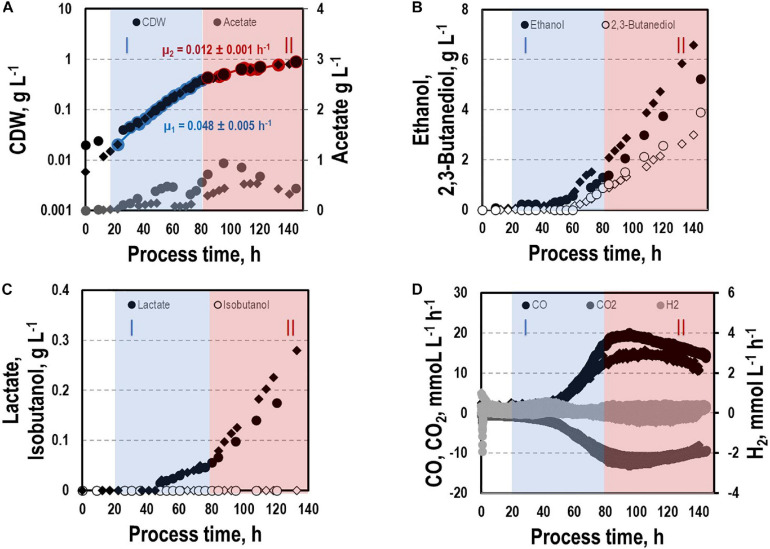
Syngas batch cultivation of CLJU[WT] in a stirred bioreactor with continuous gas supply. Depicted are concentrations of cell dry weight and acetate **(A)**, ethanol and 2,3-butanediol **(B)**, lactate and isobutanol **(C)**, and gas uptake **(D)** of two independent experiments (circles and diamonds). Two growth phases I (blue) and II (red) were identified. Statistical details are given in the Supplementary Material (Supplementary Table 1).

Before investigating a recombinant isobutanol formation based on syngas, a reference process (REF) was used to analyze growth, product formation, and substrate uptake of the *C. ljungdahlii* wildtype strain (CLJU[WT]) (Figure 2). Therefore, a syngas-based batch cultivation in a steadily gassed 3-L bioreactor was performed in duplicates. The detailed composition of the substrate gas is described in the Experimental procedures section. The growth phases, final product concentrations, and substrate-to-product yields of the process are summarized in Tables 1, 2. We identified two growth phases with μ_*exp*_ = 0.05 ± 0.005 h^–1^ [average ± standard deviation] in the first (approximately 20 – 80 h) and μ_*exp*_ = 0.01 ± 0.001 h^–1^ in the following period (approximately 90 – 120 h). After approximately 140 h, the final CDW = 0.85 ± 0.06 g L^–1^ was reached representing 2.3 ± 0.6% of totally captured carbon. Furthermore, the two growth periods were characterized by different substrate uptake patterns. During the first phase, CO uptake accompanied by proportional CO_2_ formation occurred. Maximum volumetric and biomass specific rates were *r*_*CO*_ = 16.3 ± 4.1 mmol (L^∗^h)^–1^ i.e., q_*CO*_ = 38.8 ± 4.1 mmol (g_*CDW*_^∗^h)^–1^ and r_*CO*2_ = 11.3 ± 1.4 mmol (L^∗^h)^–1^ i.e., q_*CO*2_ = 27.4 ± 3.4 mmol (g_*CDW*_^∗^h)^–1^, respectively. Subsequently, both rates decreased to *r*_*CO*_ = 12.8 ± 2.7 mmol (L^∗^h)^–1^ i.e., q_*CO*_ = 16.1 ± 3.4 mmol (g_*CDW*_^∗^h)^–1^ and r_*CO*2_ = 8.7 mmol ± 1.0 (L^∗^h)^–1^ i.e., q_*CO*2_ = 11.00 mmol ± 1.3 (g_*CDW*_^∗^h)^–1^. Only very low H_2_ uptake even revealing large deviations between the two biological replicates was observed. On average, *r*_*H*2_ = 0.2 ± 0.03 mmol (L^∗^h)^–1^ and *r*_*H*2_ = −0.01 ± 0.4 mmol (L^∗^h)^–1^ were measured for the first and second period, respectively. Acetate patterns showed similar trends in biological duplicates with acetate formation during early first phase, reaching a maximum until the beginning of the second period when consumption started. The residual of 0.38 ± 0.08 g L^–1^ represented only 0.9 ± 0.02% of totally captured carbon. On the contrary, the formation of the reduced products ethanol, 2.3-butanediol, and lactate started after the initiation of acetate formation showing a steady rise with final concentrations of 5.9 ± 0.9 g L^–1^ for ethanol, 3.5 ± 0.6 g L^–1^ for 2.3-butanediol, and 0.01 ± 0.03 g L^–1^ for lactate. Accordingly, *C ljungdahlii* converted approximately 30% of consumed carbon into reduced products. The total free Gibbs reaction energy ΔG_*R*_ of the process was – 33.55 ± 1.92 kJ C-mole^–1^.

**TABLE 1:** Maximal growth rates and final by-product concentrations of the syngas-based batch cultivations of the different *C. ljungdahlii* strains in a steadily gassed stirred bioreactor.

Strain	μ_*max*_, h^–1^	c_*CDW*_, *g* L^–1^	c_*Acetate*_, *g* L^–1^	c_*Ethanol*_, *g* L^–1^	c_2,3–BD_, *g* L^–1^	c_*Lactate*_, *g* L^–1^	c_*Isobutanol*,_ g L^–1^
CLJU[WT]	0.048 ± 0.005	0.85 ± 0.06	0.38 ± 0.08	5.90 ± 0.95	3.45 ± 0.64	0.26 ± 0.05	
CLJU[KAIA]	0.071	0.73	0.22	5.25	2.38	0.25	0.02
CLJU[KAIA]:*ilvE*	0.055	0.89	0.83	5.90	3.42	0.09	0.13

*Rates reflect exponential growth. Values of the wildtype cultivation indicate mean of duplicates.*

**TABLE 2:** Final biomass and product yields of the syngas-based batch cultivations of the different *C. ljungdahlii* strains in steadily gassed stirred-tank bioreactor (*T* = 37°C; pH = 5.9; V_*R*_ = 3 L; 500 rpm).

Strain	*Y*_*CDW*_	*Y*_*Acetate*_	*Y*_*Ethanol*_	*Y*_2,3–BD_	*Y*_*Lactate*_	*Y*_*Isobutanol*_	*Y*_*CO2*_

	C-mole_(Product)_ mole^–1^_(*CO*)_						
CLJU[WT]	0.023 ± 0.006	0.009 ± 2.3*10^–5^	0.19 ± 0.07	0.12 ± 0.002	0.007 ± 0.003		0.71 ± 0.005
CLJU[KAIA]	0.026	0.006	0.20	0.09	0.009	0.001	0.70
CLJU[KAIA]:*ilvE*	0.027	0.024	0.17	0.09	0.002	0.003	0.65

*Values of the wildtype cultivation indicate mean of duplicates.*

#### Intracellular Metabolites Pattern

**FIGURE 3:**
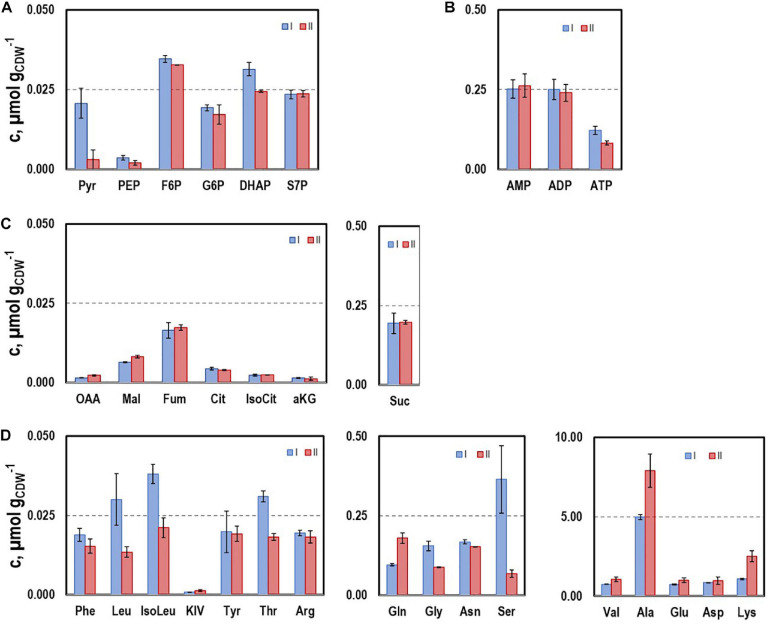
Selected intracellular pools representing Emden-Meyerhof-Parnas and the pentose-phoshate pathway **(A)**, energy metabolism **(B)**, citrate cycle **(C)**, and amino acids **(D)**. Samples were taken during growth phases I and II cultivating CLJU[WT] on syngas in a stirred tank. Error bars are derived from technical replicates.

To further characterize the physiological state of the cells 32 intracellular metabolite pools were analyzed (Figure 3) at representative sampling times. Pool sizes differed individually ranging from about 0.001 μmol g_*CDW*_^–1^ for KIV, oxaloacetate (OAA), and 2-ketoglutarate (αKG) to 8 μmol g_*CDW*_^–1^ for alanine. Whereas most pool sizes remained constant throughout the process, pyruvate and serine depleted during the second growth phase. In contrast, remarkably large pool sizes were found for valine, glutamate, aspartate, alanine, and lysine.

**FIGURE 4:**
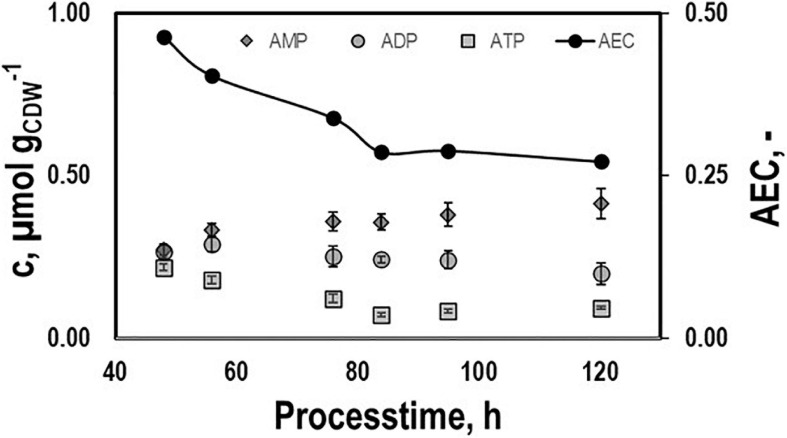
Intracellular pools of AMP, ADP, and ATP and the respective AEC values. Samples were taken during growth phases I and II cultivating CLJU[WT] on syngas in a stirred tank. Error bars are derived from technical replicates.

Intracellular patterns of ATP, ADP, AMP, and the respective adenylate energy charge (AEC) were studied to evaluate whether or not non-wanted energy shortage might exist growing on syngas (Figure 4). ATP pools dropped until they leveled out at 0.08 μmolg_*CDW*_^–1^ during the second period. A similar trend was found for AEC starting at 0.46 and ending at 0.28. On contrary, AMP and ADP rather remained at about 0.25 μmolg_*CDW*_^–1^.

### Batch Cultivations of the Recombinant Strains CLJU[KAIA] and CLJU[KAIA]:ilvE Using Syngas

#### CLJU[KAIA]

**FIGURE 5:**
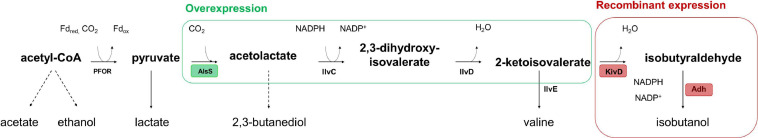
Recombinant isobutanol pathway in CLJU[KAIA] characterized by plasmid-encoded amplification of pyruvate-to-KIV conversion followed by the conversion of KIV via recombinant Ehrlich pathway. PFOR: pyruvate:ferredoxin-oxidoreductase; AlsS: acetolactate synthase; IlvC: ketol-acid reductoisomerase, IlvD: dihydroxy-acid dehydratase; Ilve: amino acid aminotransferase; KivD: ketoisovalerate decarboxalyse; Adh: alcohol dehydrogenase.

**FIGURE 6:**
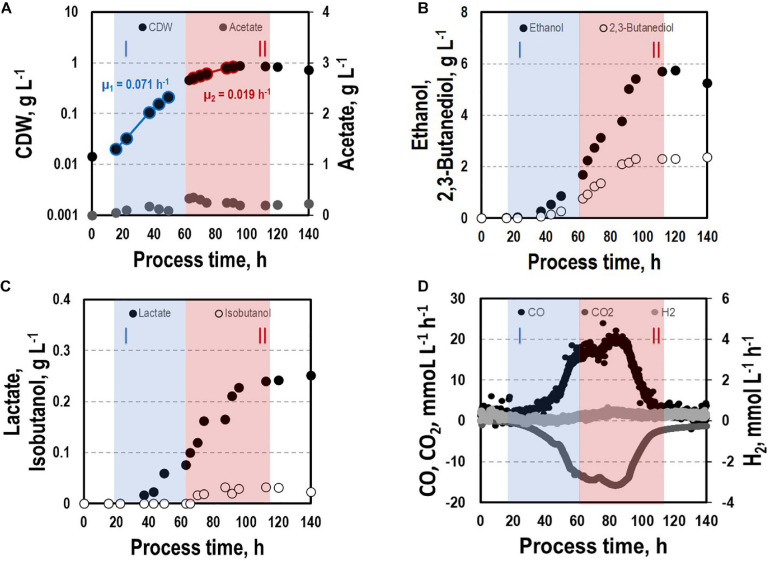
Syngas-based batch cultivation of CLJU[KAIA] in a stirred tank bioreactor with a continuous gas supply. Shown are the concentrations of cell dry weight and acetate **(A)**, ethanol and 2,3-butanediol **(B)**, lactate and isobutanol **(C)**, and the gas uptake **(D)** (*T* = 37°C; pH = 5.9; VR = 3 L; 500 rpm). Two growth phases I (marked blue) and II (marked red) were identified. Statistical details are given in the Supplementary Material (Supplementary Table 1)

Recombinant isobutanol production from syngas was studied using the strain CLJU[KAIA] which possesses plasmid-encoded amplification of pyruvate-to-KIV conversion followed by the conversion of KIV *via* recombinant Ehrlich pathway (Figure 5). Process parameters of the batch equaled those of the REF leading to kinetics and process values indicated in Figure 6 and in Tables 1, 2. Again, biphasic growth occurred revealing μ_*exp*_ = 0.07 h^–1^ between 20 – 60 h and μ_*exp*_ = 0.02 h^–1^ between 60 – 120 h. The final CDW was 0.73 g L^–1^ which equals 2.6% of the entire carbon capture in biomass. Compared to REF, CLJU[KAIA] grew 50% faster during phase I disclosing similar CO-to-CDW yield. Also, substrate uptake kinetics resembled REF showing CO_2_ formation accompanying CO uptake during phase I. Maximum volumetric and specific rates were *r*_*CO*_ = 17.6 mmol (L^∗^h)^–1^ i.e., q_*CO*_ = 34.6 mmol (g_*CDW*_^∗^h)^–1^ and r_*CO*2_ = 13.8 mmol (L^∗^h)^–1^ i.e., q_*CO*2_ = 27.0 mmol (g_*CDW*_^∗^h)^–1^. During growth period II, biomass specific rates for CO uptake q_*CO*_ = 25.2 mmol (g_*CDW*_^∗^h)^–1^ and CO_2_ formation q_*CO*2_ = 18.9 mmol (g_*CDW*_^∗^h)^–1^ decreased. Low H_2_ uptake was observed in each phase with *r*_*H*2,I_ = 0.2 mmol (L^∗^h)^–1^ followed by *r*_*H*2,II_ = 0.4 mmol (L^∗^h)^–1^. The product spectrum was similar to the REF with the final concentrations of 0.22 g L^–1^ of acetate, 5.25 g L^–1^ of ethanol, 2.38 g L^–1^ of 2.3-butanediol, 0.25 g L^–1^ of lactate, and 0.02 g L^–1^ of isobutanol. The total free Gibbs reaction energy ΔG_*R*_ of the process was – 36.15 kJ C-mole^–1^ (Table 3).

**TABLE 3:** Carbon balances and Gibb’s free reaction energies of the syngas-based batch cultivations of the different *C. ljungdahlii* strains in steadily gassed stirred-tank bioreactor (*T* = 37°C; pH = 5.9; *V*_*R*_ = 3 L; 500 rpm). Values of the wildtype cultivation indicate mean of duplicates.

Strain	C – Balance, %	Δ G_*R*_, kJ C-mole^–1^
CLJU[WT]	106.1 ± 7.6	−33.6 ± 1.9
CLJU[KAIA]	102.3	−36.2
CLJU[KAIA]:*ilvE*	96.6	−37.9

**FIGURE 7:**
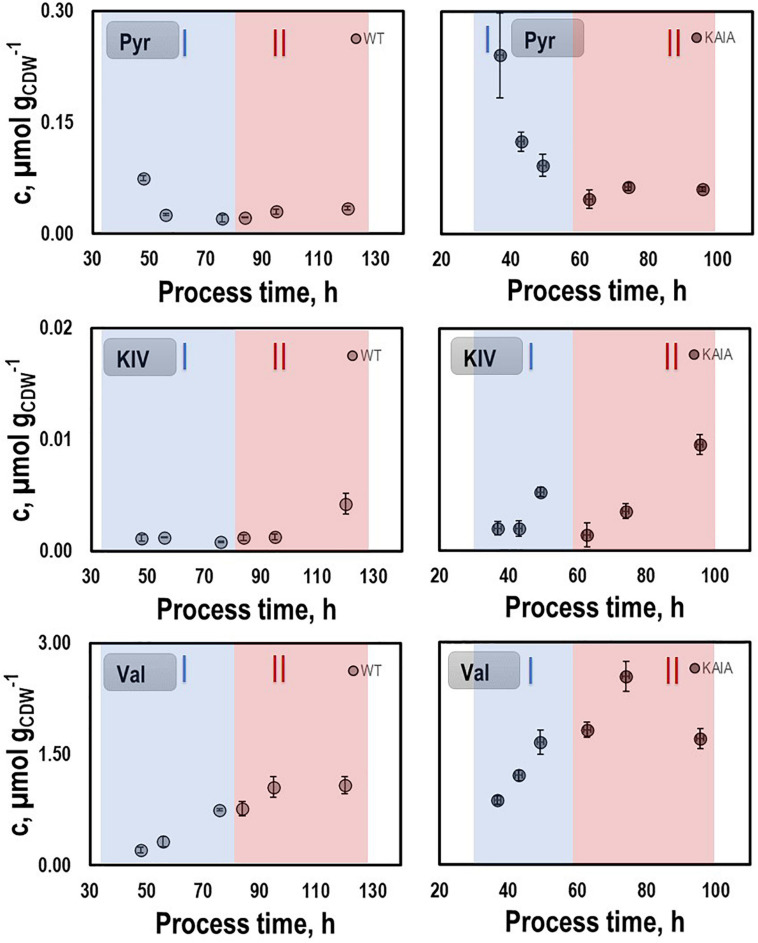
Pyruvate (PYR), ketoisovalerate (KIV), and valine (VAL) pools in autotrophic batch cultivations of CLJU[WT, REF] and CLJU[KAIA] using syngas. Error bars are derived from technical replicates.

In order to identify possible metabolic engineering targets for optimizing isobutanol production, we compared the patterns of the intracellular metabolites pyruvate, KIV, and valine with those of the REF (Figure 7). KIV courses were almost identical, notably revealing the lowest pool sizes of all. Pyruvate levels in CLJU[KAIA] were higher than in REF which might be explained by the elevated growth rate. In addition, substantially higher levels of valine were measured in CLJU[KAIA] compared to REF throughout the entire batch culture.

#### CLJU[KAIA]:*ilvE*

**FIGURE 8:**
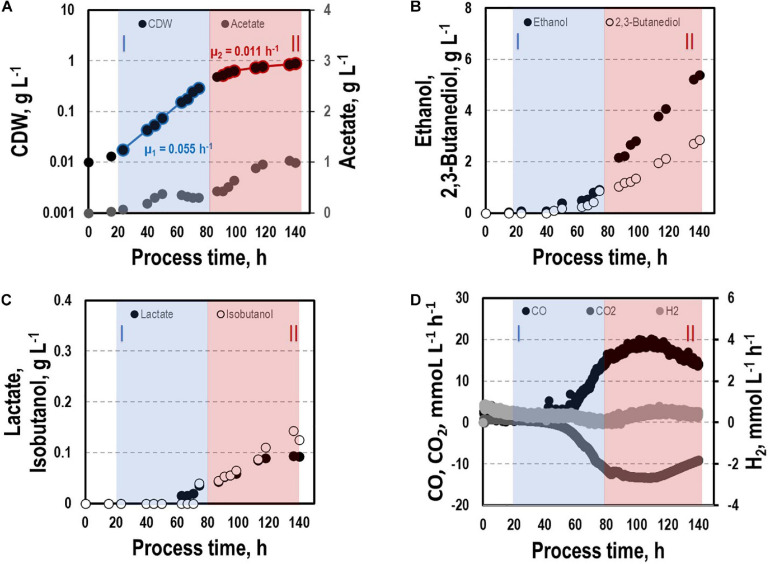
Syngas-based batch cultivation of CLJU[KAIA]:ilvE in a stirred tank bioreactor with a continuous gas supply. Shown are the concentrations of the cell dry weight and acetate **(A)**, ethanol and 2,3-butanediol **(B)**, lactate and isobutanol **(C)**, and the gas uptake **(D)** (*T* = 37°C; pH = 5.9; VR = 3 L; 500 rpm). Two growth phases I (marked blue) and II (marked red) were identified. Statistical details are given in the Supplementary Material (Supplementary Table 1).

By interrupting *ilvE* encoding valine amino transferase a block of valine synthesis was achieved. The resulting strain CLJU[KAIA]:*ilvE* was cultivated under reference conditions revealing kinetics as indicated in Figure 8 and in Tables 1, 2. Growth, substrate uptake, and product formation (except for isobutanol) resemble the wildtype. Again, we identified biphasic growth with μ_*exp,I*_ = 0.055 h^–1^ (20 – 80 h) and μ_*exp,II*_ = 0.011 h^–1^ (90 – 140 h) reaching CDW = 0.89 g L^–1^ which represents 2.7% of CO captured in biomass. Despite a suspected valine auxotrophy of CLJU[KAIA]:*ilvE* there was no reduction of growth compared to the wildtype. This indicates that the valine concentration provided by the 0.5 g L^−1^ yeast extract in the medium is sufficient. During phase I, maximum volumetric and specific rates were r_*CO*_ = 16.2 mmol (L^∗^h)^–1^ i.e., q_*CO*_ = 33.8 mmol (g_*CDW*_^∗^h)^–1^ and r_*CO*2_ = 11.9 mmol (L^∗^h)^–1^ i.e., q_*CO*2_ = 24.9 mmol (g_*CDW*_^∗^h)^–1^. The mean volumetric H_2_ uptake rate was 0.2 mmol (L^∗^h)^–1^. In phase II, biomass specific CO uptake and CO_2_ formation rates decreased to q_*CO*_ = 23.5 mmol (g_*CDW*_^∗^h)^–1^ and q_*CO*2_ = 16.1 mmol (g_*CDW*_^∗^h)^–1^. Volumetric H_2_ uptake rate slightly increased to 0.3 mmol (L^∗^h)^–1^. At the end of the process, 0.83 g L^–1^ acetate, 5.90 g L^–1^ ethanol, 3.42 g L^–1^ 2.3-butanediol, 0.09 g L^–1^ lactate, and 0.13 g L^–1^ isobutanol were determined. The total free Gibbs reaction energy ΔG_*R*_ of the process was – 37.89 kJ C-mole^–1^ (Table 3). Both, isobutanol and 2,3 butanediol formation depend on NADPH which is why internal supply was worth investigating.

### Simulation of Intracellular Flux Distribution

**FIGURE 9:**
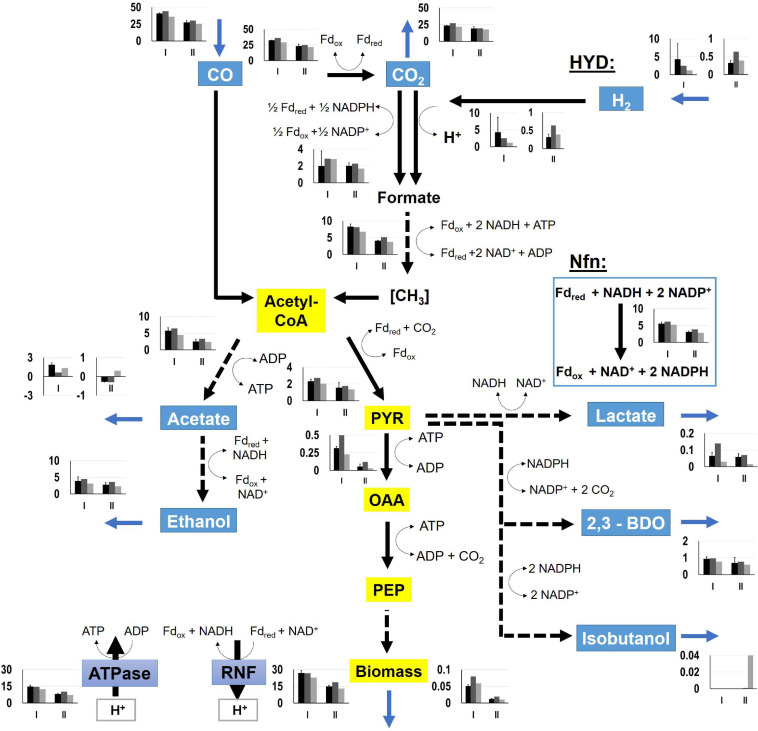
Metabolic flux distributions of CLJU[WT, REF] (black) CLJU[KAIA] (dark gray), and CLJU[KAIA]:ilvE (light gray) based on the conversion of syngas in steadily gassed batch cultivations in stirred-tank bioreactors. Illustrated are the simulated fluxes in mmol (g_CDW_*h)^–1^ for the first (I) and second (II) growth phase. Values of the wildtype cultivation indicate mean of duplicates.

For determination of NADH and NADPH availabilities in the strains, FBA were performed. Intracellular flux patterns of the “pseudo-steady states” in phases I and II were studied using the stoichiometric metabolic model “modified rSMM” (Hermann et al., 2020), that was extended by recombinant isobutanol formation (see appendix). We applied FBA as the number of the unknown intracellular fluxes exceeded the total number of measured extracellular fluxes. Since growth was exponential in both phases, we chose the maximization of biomass production as objective function in each case. Measured uptake and consumption rates further constrained the solution space. As already shown in Hermann et al. (2020) the approach allowed very well to predict real growth rates further elucidating intracellular flux patterns. Alternate application of determined metabolic flux analysis was not possible as additional measurements e.g., using 13C labeling were not accessible. Since we could not resort to this method, we restricted the solution space by using all experimentally determined uptake and secretion rates as constraints to assure that FBA results reflect real physiological states (O’Brien et al., 2015). As a prerequisite, we qualified the achieved experimental carbon closures of 106.10 ± 7.59, 100.02, and 96.55% as “sufficient” analyzing cultivations of the wild-type (REF), CLJU[KAIA], and CLJU[KAIA]:*ilvE*, respectively (Table 3). The overview of all flux patterns is given in Figure 9. Furthermore, NADH and NADPH formation related to the consumption of electron donors CO and H_2_ are listed in Table 4. Yields were derived from the WLP and from the Nfn reaction. In *C. ljungdahlii*, regeneration of NADH and NADPH are strongly intertwined and controlled by the provision of Fd_*red*_ (Hermann et al., 2020). Utilizing syngas, NADH supply is ensured *via* WLP and *via* the Rnf complex. The transhydrogenase Nfn consumes NADH providing NADPH. In turn, the reduction of CO_2_ to formate by the formate dehydrogenase activity represents a NADPH sink. Also, *C. ljungdahlii* directly reduces CO_2_ to formate utilizing H_2_ through the formate-hydrogen lyase reaction which is carried out by a complex composed of the electron-bifurcating NADP^+^- and ferredoxin-dependent [FeFe]-hydrogenase and formate dehydrogenase (Wang et al., 2013). Our simulation results show simultaneous activity of both reactions in each cultivation. However, the share of the formate-hydrogen lyase-like reaction strongly decreases due to limited H_2_ uptake during phase II. This finding is remarkable as the explicit use of this formate-hydrogen lyase-like reaction for CO_2_ reduction was found in continuously cultivated *C. autoethanogenum* (Valgepea et al., 2017). Apparently, *C. ljungdahlii* adapts CO_2_ reduction to current needs via flexible enzyme activities of said reductive route. Interestingly enough, NADPH yields were up to 30% higher in isobutanol producers than in the wildtype. Higher activities of transhydrogenase (Nfn) and of the formate-hydrogen reaction rate enable this phenotype. Nevertheless, strongly decreasing NADPH yields of 65 – 70% were determined in the second growth phase for each cultivation. On the contrary, NADH availabilities almost remained constant during phases I and II, only showing a slight 20% decrease for CLJU[KAIA]:*ilvE*.

**TABLE 4:** NADH and NADPH yields derived from flux balance analysis for the first (I) and second (II) growth phase considering the WLP and the Nfn reaction.

Strain	Phase I		Phase II	
	NADH, mole mole_*CO* + *H2*_^–1^	NADPH, mole mole_*CO* + *H2*_^–1^	NADH, mole mole_*CO* + *H2*_^–1^	NADPH, mole mole_*CO* + *H2*_^–1^
CLJU[WT]	0.119 ± 0.026	0.020 ± 0.001	0.131 ± 0.028	0.006 ± 0.001
CLJU[KAIA]	0.095	0.029	0.147	0.01
CLJU[KAIA]:*ilvE*	0.109	0.027	0.086	0.009

*Values of the wildtype cultivation indicate mean of duplicates.*

## Discussion

### Syngas-Based Reference Process

Metabolizing syngas during a batch cultivation *C. ljungdahlii* shows a biphasic growth behavior, that is characterized by growth reduction with simultaneously increased formation of reduced products. This coincidence was also observed in a two-stage process cultivating *C. ljungdahlii* on syngas (Richter et al., 2016). Previous studies have shown that shifts from acetogenesis to solventogenesis in syngas-fermenting *C. ljungdahlli* are accompanied by growth reduction. Metabolic rearrangements may be induced by pH shifts, nutrient limitation or addition of reducing agents into the growth medium (Gaddy and Clausen, 1992; Cotter et al., 2009; Mohammadi et al., 2016; Richter et al., 2016). In this context, Valgepea et al. (2017) demonstrated that solvent formation in *C. autoethanogenum* may be promoted by simply increasing biomass concentrations. This can be explained by the metabolic link between energy conservation and redox management in acetogens. Shifting to solventogenic alcohol production improves ATP availability *via* the Rnf-ATPase-system (Richter et al., 2016; Valgepea et al., 2017; Hermann et al., 2020). Further evidence was given by Oswald et al. (2016). They measured CO uptake rates of 33 – 100 mmol (g_*CDW*_^∗^h)^–1^ and CO_2_ production rates of 13 – 40 mmol (g_*CDW*_^∗^h)^–1^ during a batch-cultivation of *C. ljungdahlii* with continuous gas supply. These values fit fairly well to our study. In contrast to our process, however, they identified a simultaneous and equivalent uptake of CO and H_2_. The simultaneous utilization of these gases is also shown in further studies describing continuous cultivations performed in chemostat mode installing 0.04 h^–1^ dilution rate (Richter et al., 2013; Martin et al., 2016; Valgepea et al., 2017, 2018). Our observations did not reveal proportionality between CO and H_2_ uptake which might be explained by the different syngas composition used (32.5% H_2_, 32.5% CO, 16% CO_2_, 19% N_2_). H_2_ uptake and the product spectrum depend on the H_2_/CO ratio (Jack et al., 2019) which was 1 in Oswald et al. (2016) and 0.54 in this study. CO is known to be a strong inhibitor of the hydrogenase activity (Jones and Woods, 1986; Goldet et al., 2009; Devarapalli et al., 2016), but CO utilization yields the formation of more reduced products (Jack et al., 2019; Hermann et al., 2020). Moreover, acetate formation is favored by increased H_2_ consumption (Jack et al., 2019). Hence, the ratio of H_2_/CO affects the product portfolio strongly.

Intracellular metabolites pattern during the process reveals a clear depletion of pyruvate accompanied by a strong accumulation of alanine in the second growth phase. Pyruvate serves as key precursor for 2,3-butanediol and lactate, which are mainly formed in the second phase of the process. In this regard, pyruvate depletion may limit the formation of 2.3-butanediol and isobutanol. Notably, the decreasing L-serine pool might hint to *C. ljungdahlii’s* capacity converting L-serine to pyruvate via L-serine dehydratase to refill the pyruvate pool (Köpke et al., 2010).

Alanine may be synthesized by decarboxylation of aspartate and by transamination of valine, glutamate, and pyruvate (Parker and Pratt, 2010). Accordingly, it may serve as carbon and nitrogen storage that could be easily converted to pyruvate. The product formation of *C. ljungdahlii* is driven by its energy and redox management (Hermann et al., 2020). To counteract a surplus of reducing equivalents caused by continuous uptake of CO, *C. ljungdahlii* needs to produce more ethanol and 2.3-butanediol during the phase of retarded growth (Richter et al., 2016; Hermann et al., 2020). In addition, Richter et al. (2016) postulated that the metabolic shift from acidogenesis to solventogenesis of *C. ljungdahlii* is not regulated at the proteome level but rather by thermodynamics. Therefore, we hypothesize that the intracellular accumulation of alanine, valine, and glutamate enables *C. ljungdahlii* to react flexibly on increasing demands for pyruvate, the “doorman” metabolite for getting rid of “surplus” electrons. This hypothesis is supported by the intracellular AEC and AXP patterns. Despite a reduction of AEC and the ATP concentration in the first growth phase, both values remained constant in the following period.

The initial decrease of the ATP pools may be associated with anabolic ATP needs considering that acetogens are living at the edge of thermodynamic feasibility. Retarded growth, consumption of acetate, and enhanced formation of reducing products enable *C. ljungdahlii* to keep its ATP pool constant during the second period. This may be advantageous for the recombinant production of isobutanol. Schatschneider et al. (2018) measured 0.1 μmolg_*CDW*_^–1^ ATP, 0.09 μmolg_*CDW*_^–1^ ADP, and 0.95 μmolg_*CDW*_^–1^ AMP in CO-consuming, steadily growing *C. autoethanogenum*, a close relative of *C. ljungdahlii*. Those values lead to the AEC of 0.13 which fit to the observations of this study. Interestingly, AXP levels and AEC are much lower than the so-called “physiological” levels that range from 0.80 to 0.95 (Chapman et al., 1971). However, the latter rather mirror heterotrophic growth under aerobic conditions with ATP/C ratios of 6.3 – 2.3 (assuming catabolism of glucose with P/O ratios of 2.0 – 1.1). Instead, anaerobic ATP/C gain under autotrophic growth is an order of magnitude lower (Hermann et al., 2020).

### Recombinant Isobutanol Formation

Syngas-based recombinant isobutanol formation by CLJU[KAIA] was successfully achieved, although at a low level, still. Given that the total free Gibbs reaction energy ΔG_*R*_ was even lower than in REF (Table 3), no energy limitation was anticipated. Remarkably higher levels of valine compared to the wildtype cultivation together with the very low pyruvate levels suggested to block valine synthesis for increasing isobutanol formation. By this, a 6.5-fold increase of isobutanol titer compared to CLJU[KAIA] was achieved which supports the findings of Weitz et al. (2021). Energetically, the process should have been well equilibrated (Table 3) although intermediary shortcomings of reducing equivalents may not be ruled out completely. However, FBA revealed a limitation of NADPH at the end of the process, while NADH availabilities almost remained constant. Consequently, further improvements of isobutanol formation using CLJU[KAIA]:*ilvE* should be achievable by replacing the cofactor dependency on NADPH by NADH. Using a NADH-dependent variant of the ketol-acid reductoisomerase Weitz (2020) demonstrated increased isobutanol formation (18%). Ethanol and 2,3-butanediol are the main products during syngas-based batch fermentation of *C. ljungdahli* applying a H_2_/CO ratio of approximately 0.5 (Table 1). Further strain optimization may aim to detour said reductive power into isobutanol formation. In case of ethanol this goal is very challenging as ethanol may be considered as a vital by-product of the acetaldehyde:ferredoxin oxidoreductase (AOR) which links its formation with ATP synthesis (Mock et al., 2015; Liew et al., 2017; Hermann et al., 2020; Liu et al., 2020; Zhu et al., 2020). However, elimination of 2,3-butanediol production may increase isobutanol formation as both products originate from the precursor pyruvate requiring NADPH as electron donor. Unfortunately, a first attempt to eliminate 2,3-butanediol formation in *C. ljungdahlii* was not successful. Thus, further approaches need to be performed (Weitz et al., 2021). Additionally, improved supply of H_2_ may also increase the isobutanol formation. To check the hypothesis processes with higher H_2_/CO ratios and/or higher reactor pressures during the solventogenic phase could be performed.

## Conclusion

*Clostridium ljungdahlii* is well equipped to convert syngas mixtures with H_2_:CO ratios of 0.5 into reduced products. 30% of totally consumed carbon were used to produce mostly ethanol and 2.3-butanediol. Predominately, reducing equivalents originate from CO. However, the additional H_2_ uptake, even if low, enables *C. ljungdahlii* to adapt simultaneous CO_2_ reduction flexibly to NADPH needs. Intracellular pyruvate availability turned out to be a carbon bottleneck of alcohol formation. The implementation of the Ehrlich-pathway partly alleviated the carbon shortage. However, only the additional block of valine formation enabled to harvest the available carbon which resulted in a 6.5-fold increase of isobutanol formation. Analyzing the redox condition in CLJU[KAIA]:*ilvE* gives rise to the conclusion that a novel metabolic engineering target should be addressed next: The increased supply of NADPH. Targeting this goal should be in the focus of future autotrophic isobutanol formation with engineering *C. ljungdahlii*.

## Data Availability Statement

The raw data supporting the conclusions of this article will be made available by the authors, without undue reservation.

## Author Contributions

MH designed the study, conducted the bioreactor experiments, reconstructed the stoichiometric model, performed flux balance analyses, analyzed the datasets, drafted the manuscript, and supported the laboratory conversion. AT and MH designed and performed the metabolomics analysis. AF designed and set up the laboratory for the performance of synthesis gas bioreactor studies. AN advised the network reconstruction and flux balance analysis. SW constructed the recombinant strains CLJU[KAIA] and CLJU[KAIA]:*ilvE*. FB and PD supervised the strain reconstruction and advised the study. RT conceived the study and corrected the manuscript. MH, AT, SW, FB, PD, and RT read and approved the final manuscript. All authors contributed to the article and approved the submitted version.

## Conflict of Interest

The authors declare that the research was conducted in the absence of any commercial or financial relationships that could be construed as a potential conflict of interest.
