# Supplementary material for: Identifying and Engineering Bottlenecks of Autotrophic Isobutanol Formation in Recombinant C. ljungdahlii by Systemic Analysis
Source: Front Bioeng Biotechnol. 2021 Mar 3;9:647853. doi: 10.3389/fbioe.2021.647853 (PMC7968104; doi:10.3389/fbioe.2021.647853)
Supplement: Supplementary file 1 [file Data_Sheet_1.pdf]

Supplementary Material

Supplementary Material (.pdf file) contains:

- Supplementary Table S.1: Determination of the exponential growth rate for each data set and growth phase observed using regression fitting. Summarized are the determined growth rates μ and the corresponding coefficients of determination R^2 for the cultivation experiments with the three strains CLJU[WT, REF], CLJU[KAIA] and CLJU[KAIA]::*ilvE*

- Supplementary Figure S.1: Comparative analysis of growth rates μ (**A**) and CO uptake rates q_{CO} (**B**) for the syngas-based batch cultivation of CLJU[WT, REF], CLJU[KAIA] and CLJU[KAIA]::*ilvE* in a stirred tank bioreactor with a continuous gas supply. Error bars are derived from biological duplicates for the cultivation of CLJU[WT, REF].

- Experimental procedure

Supplementary Information (.xlsx file) contains:

- Sheet tab 1: modified_rSMM_compounds

- Sheet tab 2: modified_rSMM_reactions

- Sheet tab 3: flux simulations

- Sheet tab 4: delta G_R determination

Table S1: Determination of the exponential growth rate for each data set and growth phase observed using regression fitting. Summarized are the determined growth rates μ and the corresponding coefficients of determination R^2 for the cultivation experiments with the three strains CLJU[WT, REF], CLJU[KAIA] and CLJU[KAIA]::*ilvE*

**Results**	**CLJU[WT] I**		**CLJU[WT] II**		**CLJU[KAIA]**		**CLJU[KAIA]::ilve[KAIA]**	
	**Phase 1**	**Phase 2**	**Phase 1**	**Phase 2**	**Phase 1**	**Phase 2**	**Phase 1**	**Phase 2**
**μ. h-1**	0.044	0.012	0.052	0.013	0.072	0.020	0.055	0.011
**R^2. -**	0.998	0.969	0.992	0.993	0.996	0.994	0.999	0.945

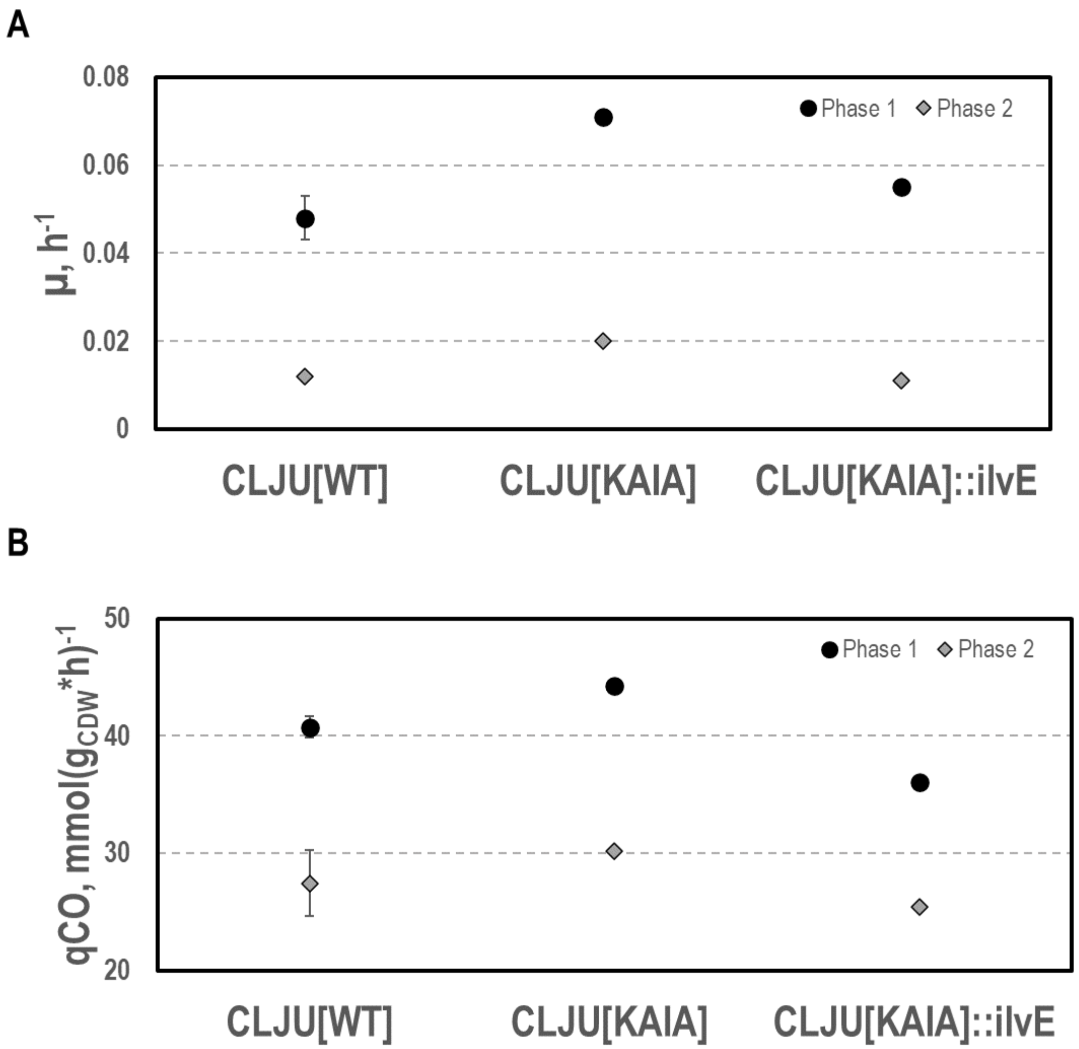

Figure S.1: Comparative analysis of growth rates μ (**A**) and CO uptake rates q_{CO} (**B**) for the syngas-based batch cultivation of CLJU[WT, REF], CLJU[KAIA] and CLJU[KAIA]::*ilvE* in a stirred tank bioreactor with a continuous gas supply. Error bars are derived from biological duplicates for the cultivation of CLJU[WT, REF].

Experimental procedure

Bacterial strains, growth medium and pre-culture preparation

C. ljungdahlii DSM 13528 (Tanner et al., 1993) was obtained from the German Collection of Microorganisms and Cell Culture (DSMZ). The recombinant C. ljungdahlii strains CLJU[KAIA] and CLJU[KAIA]::Ilve[KAIA] were kindly provided by the group of Dr. Frank Bengelsdorf (Institute of Microbiology and Biotechnology, University Ulm). Tanner mod. PETC medium (ATCC medium 1754) with 15 g L-1 MES buffer and 0.5 g L-1 yeast extract was used for pre-culture bioreactor experiments. The redox indicator resazurin, however, was solely used for the preculture steps. The preculture seed train contained several heterotrophic and autotrophic cultivation steps is precisely described by Hermann et al. (2020). Briefly, starting from a frozen cell stock two heterotrophic preculture steps in the scales of 5 mL and 50 mL were performed. An additional syngas-based preculture step was used to adapt the cells to the fructose-free medium prior to the inoculation of the bioreactor.

Batch cultivation studies in a stirred-tank reactor with different substrates

Anaerobic syngas-based batch cultivations were performed in a fully controlled 3 L stirred-tank bioreactor (Bioengineering, Wald, Switzerland) with an operational volume of 1.5 L. The detailed reactor equipment was previously described in Hermann et al. (2020). Temperature and pH were kept constant at 37 °C and 5.9 respectively. The agitation spees of the impeller was 500 rpm during the whole cultivation process. The substrate gas was fed continuously to the reactor using one mass flow controller (Bronkhorst High-Tech B.V., Ruurlo, Netherlands) and a predefined gas mixture with a constant gassing rate of 13.2 L h-1. The gas composition was 55% CO, 30% H2, 5% CO2 and 10% Ar. To set anaerobic conditions, the medium containing bioreactor was sparged with nitrogen with a gassing rate of 60 L h−1 applied for 2 h. Off-gas measurements guaranteed that oxygen concentrations were always below 0.01% (vv−1). Afterwards, the medium was equilibrated with the substrate gas for 5 h. Two hours prior to inoculation of the bioreactor, sterile reducing agent was added (Tanner et al.,1993). To observe growth, extracellular product formation and intracellular metabolite pools, samples were taken frequently during the cultivations.
Reproducibility for all fermentation process was demonstrated by performing the wildtype reference process in bioduplicates. Cultivation processes of the recombinant strains were not replicated.

Analytical methods

Biomass concentration analysis

Cell density was determined offline via a UV/Visible spectrophotometer (Ultrospec 1100 pro, Amersham Bioscience GmbH, Freiburg, Germany) at 600 nm. Thereof, the CDW concentration in [gCDW L-1] was estimated according to a previously determined CDW/OD600nm – correlation factor of 0.25 gCDW L-1 (OD)-1 for all analyzed C. ljungdahlii strains. To prepare the CDW pellets three 4 mL samples of the cell-suspension were taken at each sampling time, washed twice with mineralized water, centrifuged at 6900 rcf and 4 °C

for 5 min (5430 R, Eppendorf, Hamburg, Germany) and afterwards transferred to pre-weighed glass vials with a total volume of 1.5 mL. The cell pellets were then dried at 105 °C for at least 24 h in a convection oven (Heraeus, Hanau, Germany). Empty glass vials as well as the cell pellets were stored in a desiccator after cooling (Duran vacuum desiccator, DWK Life Sciences GmbH, Mainz, Germany) for several hours before weighting..

Analysis of extracellular products

The extracellular formation of ethanol, acetate, 2,3-butanediol, lactate, and isobutanol was observed using an isocratic high-performance liquid chromatography (HPLC) equipped with a RI detector and a Rezex ROA-Organic Acid H^+ column. Measuring parameters and sample preparation are described in Hermann *et al*. (2020).

LC-MS based analysis of intracellular metabolites' concentrations

Intracellular metabolites' concentrations in [µmol gCDW-1] were quantified using an HPLC system (1200 Series, Agilent, Santa Clara, CA, USA) coupled to a triple quadrupole tandem mass spectrometer (QQQ-MS/MS) equipped with an electrospray ion source (Agilent 6410B, Agilent Technologies, Waldbronn, Germany). The quantification method for non-derivatized polar metabolites was based on a bicratic zwitterionic hydrophilic interaction chromatography (ZIC-pHILIC) with alkaline (pH 9.2) mobile phase conditions (Teleki et al., 2015). To detect the metabolites a negative (ESI-) ionization and multiple reaction monitoring (MRM) mode with pre-optimized precursor-to-product ion transitions were used (Teleki et al., 2015). Metabolite standards were obtained from Sigma-Aldrich (Schnelldorf, Germany). MS-grade water, methanol, chloroform and acetonitrile were purchased from Carl Roth (Karlsruhe, Germany). Metabolite standard stock-solutions were prepared in MS-grade water and stored at -70 °C. For sample preparation an adapted sequential protocol via fast centrifugation treatment (FCT) (Plassmeier et al., 2007) and a subsequent cold methanol-chloroform extraction (CME) was applied (Koning and Dam, 1992). Therefore, 5 mL cell suspension each were taken periodically as triplicates in the course of the exponential growth phases of the batch cultures. Afterwards the samples were centrifuged at 6900 rcf and -11 °C for 2 min (5430 R, Eppendorf, Hamburg, Germany) and subsequently washed with 5 mL ice-cold isotonic 0.9 % (vv-1) sodium chloride solution. Resulting cell pellets and cultivation supernatants (analogues extracellular samples) were immediately quenched by liquid nitrogen and temporarily stored at -70 °C.

For the extraction procedure the cell pellets were resuspended in 200 µL of a pre-cooled 50 % (vv-1) methanol solution using pulse vortexing (20 s) and mixed with 200 µL of pre-cooled chloroform. These extracts were incubated for 1.5 h at -20 °C and subsequently shaken for 1 h at room temperature using a cellmixer (CMV, Labortechnik Fröbel GmbH, Lindau, Germany). Afterwards the samples were centrifuged at 18000 rcf at -11 °C for 15 min (5430 R, Eppendorf, Hamburg, Germany) and the upper aqueous methanol phase was removed and temporarily stored at -70 °C until the LC-MS measurement.

Directly before analysis 10 mM ammonium acetate, 60 % (vv-1) acetonitrile, 2.5 mM 2-keto-3-deoxy-6-phosphogluconate (KDPG) and 8 % (vv-1) uniformly labeled (U-13C) Corynebacterium glutamicum metabolite extracts (Feith et al. 2019) were added to samples and standards. KDPG was used for monitoring instrumental fluctuations. U-13C-labeled metabolites, however, were crucial for the absolute quantification

of metabolites accounting for matrix effects. To absolutely quantify the metabolites a standard-based external calibration using isotope dilution mass spectrometry (IDMS) was used. Therefore, an external calibration range (2.5 nM – 40 µM) with 19 levels was developed based on previous measurements. Linear regression of ratios of non-labeled metabolite peak areas and U-13C-labeled analogues plotted against the respective concentration levels were used as external calibration curves.

Due to their high reactivity, the analysis of α-keto acids (aKG, Pyruvate and OAA) required a preceding derivatization treatment based on the condensation of aldehyde and keto groups by phenylhydrazine (Zimmermann et al., 2014). In addition, a quantification method based on bicratic reverse phase chromatography (RPLC) with acidic mobile phase conditions was applied. For this purpose, an adapted derivatization strategy as well as the respective LC-MS/MS protocol was developed and described by Junghans et al. (2019). Therefore, to determine the intracellular pools of pyruvate, KIV, aKG and OAA 2.5 µL of a freshly prepared 50 mM phenylhydrazine stock solution was added to 24 µL to the metabolite extracts. Additionally, the samples were spiked with 4 µL of a defined standard mix or water and mixed with 1 µL of a 2.2 mM glyoxylate (Gxy) solution. After an incubation at room temperature for 1h the samples were quenched with 0.5 µL of a 10%(vv-1) formic acid stock solution and 18 µL of acetonitrile. Gxy was considered to monitor instrumental fluctuations and the standard mix was needed for the absolute quantification of the respective α-keto acids. Based on previous measurements the composition of the standard mix was set with 12 µM pyruvate, 1.6 µM OAA, 2 µM KIV and 6 µM aKG. By means of different water to standard mix ratios during sample preparation internal calibration curves with four levels for each metabolite were achieved. By this, internal calibration curves resulted from a standard quadruple addition of defined amounts of the respective metabolite standards directly to the sample.

Online analysis of the exhaust gas

Exhaust exhaust gas measurement was performed online by mass spectrometry (Prima Pro, Thermo Fischer Scientific, Waltham, USA). By means of the gas concentrations in the inlet and outlet gas streams and the adjusted inlet flow rate the differences in the molar CO, CO2 and H2 concentrations during the cultivations were determined (formula 1). In each case Argon was used as inert gas. The volumetric rates ri(t) were integrated over time to calculate the molar concentrations of the gases produced or consumed by the cell at defined time points of the process. To account for dissolved CO2, total inorganic carbon (TIC) was measured in cell-free samples as described in Graf et al., (2018). Therefore, a TC analyzer (Multi N/C 2100s, Analytik Jena, Jena, Germany) was used.

$$r_i\,(t) = \frac{p}{R * T} * \frac{V_{g,in}}{V_R} * \left(\frac{c_{i,in}}{100} - \frac{c_{Ar,in}}{c_{Ar,out}} * \frac{c_{i,out}}{100}\right) \tag{1}$$

Ri (t) reflects the volumetric uptake rate of the respective gas at one time point of the cultivation process. Normal conditions were assumed to estimate the values of the pressure p, the temperature T and the gas constant R. Vg,inlet is the aeration rate and VR represents the working volume of the reactor..

Determination of cell specific rates

Biomass-specific substrate uptake and product formation rates were calculated by considering the exponential growth rate µ, the biomass substrate yield $Y_{X/S}$ or the biomass product yield $Y_{X/P}$ respectively (formula 2 and 3). In prior the exponential growth rate µ was estimated graphically by a linear regression of logarithmic CDW curves as function of the process time. The biomass substrate yields as well as the biomass product yields were also determined graphically a linear fit of the substrate or product concentration curves as function of the biomass concentration was applied.

$$q_S = \frac{\mu}{Y_{X,S}} \qquad (2)$$

$$q_P = \frac{\mu}{Y_{X,P}} \qquad (3)$$

Determination of the Gibbs free energy changes ΔG_R

Gibbs free reaction energy changes ΔG_R were calculated to compare the individual processes at the energetic level as described by Villadsen *et al*. (2011). Corresponding calculations for the individual processes are summarized in supplementary information (tab sheet 4).

Flux Balance Analysis (FBA)

Model simulations were performed based on the Insilico DiscoveryTM platform using the previously reconstructed and described model rSMM (Hermann et al., 2020), which was supplemented by a formate-H2 lyase reaction (Wang et al., 2013). This model is characterized by a constant growth-associated maintenance (GAM) value of 46.7 mmol ATP gCDW-1 (Nagarajan et al., 2013) and the invariable non-growth-associated maintenance value (NGAM) of 5 mmol (gCDW*h)-1. For NGAM estimation the mean maintenance cost identified for the closely related acetogen C. autoethanogenum growing on different gaseous substrates was considered (Valgepea et al., 2018; Heffernan et al., 2020). Further assumption were: (i) The ATP synthase reaction has a conservative stoichiometry of 3.66 H+ per 1 ATP (Mock et al., 2015); (ii) the hydrogenase is an electron-bifurcating, NADP+ and ferredoxin-dependent enzyme that refers to 2 H2 for the reversible reduction of NADP+ and Fdox (Mock et al., 2015); (iii) the formate dehydrogenase reaction uses NADPH and Fdred as electron donor

(Schuchmann and Muller, 2014); (iv) using the membrane-bound Rnf complex 2 protons are translocated via the Fdred:NAD+ oxidoreductase reaction (Perez et al.,2013); and (v) the methylene-H4F reductase reaction catalyses the coupled reduction of methylene-H4 and ferredoxin Fdox with NADH (Köpke et al., 2010; Mock et al., 2015). Furthermore, the model is characterized by 117 intracellular and 46 transport reactions thereby balancing 180 metabolites (inner degree of freedom: 12, outer degree of freedom: 22). As the degree of freedom exceeds the maximal number of quantifiable fluxes, flux balance analysis was used to investigate the intracellular flux distribution (Schilling et al., 2000; Orth et al., 2010). Maximization of biomass production was set as objective function, while all experimentally determined product formation and substrate uptake rates were used to constrain the solution space (O'Brien et al., 2015). Due to the reversibility of the phosphotransacetylase (PTA), the acetatekinase (ACK), aldehyde oxidoreductase (AOR) and the acetaldehyde/alcohol dehydrogenase (ADH) reactions a biased ATP formation may occur caused by futile cycling in the acetate and ethanol formation paths. To prevent this a two-step simulation was performed. First a maximum acetyl-CoA synthase flux (rACCOAS) was estimated based on the experimentally observed substrate uptake rates. RACCOAS was subsequently used to limit fluxes of the PTA and ACK reaction, respectively. The total organic carbon content of the 0.5 g L-1 yeast extract present in the cultivation medium accounted for less than 5% of the total incorporated carbon during all cultivations we described. Therefore, yeast extract was not considered for modelling and carbon balancing. Carbon balances were calculated by dividing the sum of C-mol products by the sum of C-mol substrates. For biomass a molecular weight of 24 g mol-1 was assumed. In case of carbon recoveries > 100% the sum of the product fluxes to the sum of substrate fluxes (set to 100%) was normalized to analyze the relative distributions.
